# Coffee Consumption and Cystatin-C-Based Estimated Glomerular Filtration Rates in Healthy Young Adults: Results of a Clinical Trial

**DOI:** 10.1155/2011/146865

**Published:** 2011-06-16

**Authors:** Masafumi Saito, Tohru Nemoto, Satoshi Tobimatsu, Midori Ebata, Yulan Le, Kei Nakajima

**Affiliations:** Division of Clinical Nutrition, Department of Medical Dietetics, Faculty of Pharmaceutical Sciences, Josai University, 1-1 Keyakidai, Sakado, Saitama 350-0295, Japan

## Abstract

Recently it has been reported that the estimated glomerular filtration rate (eGFR) is higher in habitual coffee consumers than in noncoffee consumers. However, the causality remains unclear. Therefore, we conducted a clinical trial to investigate the effects of coffee consumption on kidney function. Nineteen asymptomatic nonsmokers aged 21–27 years old participated in this study. They consumed coffee (18 g coffee beans/450 mL per day) or green tea as a comparator for 2 weeks in a crossover design. Although creatinine-based eGFR was not affected after consuming either beverage, all cystatin-C-based eGFRs determined using five different equations were significantly increased after coffee consumption (means: 5.0–7.7%), but not after green tea consumption (means: 0.1–1.6%). Serum adiponectin and magnesium levels increased significantly after coffee consumption (means: 13.6% and 4.3%, resp.), but not after green tea consumption. These findings suggest that even a short period of coffee consumption may increase cystatin-C-based eGFR, along with favorable changes in serum adiponectin, in healthy young adults.

## 1. Introduction

Coffee is one of the most frequently consumed beverages worldwide. Habitual coffee consumption has been putatively associated with some benefits, including prevention of type 2 diabetes and cardiovascular diseases [1–5]. In turn, these disorders show very strong associations with impaired kidney function. We previously reported that, in apparently healthy people, habitual coffee consumption is associated with normal or increased estimated glomerular filtration rate (eGFR) determined based on serum creatinine levels [6]. This finding was recently supported by a cross-sectional study of Japanese individuals [7] in which the authors also found that the association was independent of inflammation assessed by circulating C-reactive protein and was independent of sugar consumption. However, because of the nature of cross-sectional studies, the cause-effect relationship remains unclear. Therefore, to elucidate the causality and possible underlying mechanism is of scientific interest and important in terms of public health, because the prevalence of chronic kidney disease, including diabetic nephropathy, is increasing worldwide [8].

Therefore, we conducted a clinical trial to investigate the effects of coffee consumption on kidney function in healthy young adults. In this study, kidney function was assessed by eGFR based on the serum creatinine concentration and on the serum concentration of the low molecular weight protein cystatin C. The latter is sensitive to kidney function independently of muscle mass, particularly in people with mildly impaired kidney function [9–11]. We selected green tea as a control because it is a common beverage among Japanese individuals and because green tea consumption has also been reported to be inversely associated with type 2 diabetes in several studies [2, 4, 5, 12]. It has also been shown that serum adiponectin increases after coffee consumption [13, 14] and that coffee contains plenty of magnesium. Therefore, we examined serum adiponectin and magnesium levels to evaluate these effects on eGFR.

## 2. Methods

This study was approved by the Ethics Committee of Josai University and by our departmental ethics committee. We randomly recruited 19 apparently healthy nonsmokers aged 21–27 years old (8 men and 11 women). They had no self-reported medical history of cardiovascular diseases or kidney diseases such as glomerulonephritis. All participants gave written informed consent.

In April and May 2010, they consumed coffee (MJB Basic Blend, Tokyo, Japan, 18 g coffee beans/450 mL per day) or green tea (STANCUP, Tokyo, Japan, 6 g tea leaf/450 mL per day), equivalent to three cups per day, for 14 consecutive days in a crossover design. Coffee and green tea was prepared using coffee machines and tea bags, respectively, at a meeting room at the university by our staff every morning. Subjects were given a thermos containing 450 mL of coffee or green tea in the morning and drunk it without milk or sugar during the day. The participants followed an 8-day washout period before participating in the study and a 7-day washout period between each intervention. Throughout the study, including the washout periods, the participants were required to refrain from exhausting exercise and drinking alcohol, coffee, or tea, other than that provided.

At baseline, the subjects were randomly divided into two groups. Participants in one group (*n* = 10, men/women, 5/5) received coffee in the first phase and green tea in the second phase, while the other group (*n* = 9, men/women, 3/6) received green tea in the first phase and coffee in the second phase.

Anthropometric measurements, blood pressure tests, and laboratory tests were conducted in the early morning at the same time of the day. Clinical and biochemical variables were measured with standard methods using autoanalyzers. The serum creatinine concentration was measured enzymatically (Shika liquid-S, CRE, Kanto Chemical Co., Inc., Tokyo, Japan). Serum adiponectin and serum magnesium were measured using a human adiponectin enzyme-linked immunosorbent assay (Otsuka Pharmaceutical Co., Ltd., Tokyo, Japan) and a colorimetric method (Daiichi Fine Chemical Co., Ltd., Tokyo, Japan), respectively. Intra-assay coefficients of variation for creatinine, adiponectin, and magnesium were <5%, <10%, and <3.0%, respectively. Serum cystatin C was measured by the latex agglutination turbidimetry method (Auto Cystatin C-BML Inc., Tokyo, Japan). The reference range, as described in the manufacturer's instructions, was 0.40–0.91 mg/L. According to the report by Wada et al. [15], who measured serum cystatin C using the same method used in our study, the intra-assay and interassay coefficients of variation were 0.55–0.81% and 0.48–1.27%, respectively. 

HbA1c was measured in Japan Diabetes Society (JDS)—HbA1c units by high-performance liquid chromatography. HbA1c was converted to National Glycohemoglobin Standardization Program (NGSP) levels by the formula HbA1c (%) (NGSP) = HbA1c (JDS) (%) + 0.4%, considering the relational expression of HbA1c (JDS) (%) measured by the previous Japanese standard substance and measurement methods [16].

Kidney function, as assessed by eGFR, was calculated using the following equations. The serum creatinine-based eGFR was determined based on a modified version of that used in the Diet in Renal Disease study for Japanese subjects [17]:

(1) $$\begin{array}{cc} & \text{eGFR}\;\left(\text{mL/min/}1.73\;{\text{m}}^{2}\right)\\ & \;\;=194\times {\text{Cr}}^{-1.094}\times {\text{Age}}^{-0.287}\;\left(\text{if female}\right)\times 0.739, \end{array}$$

Cr = serum creatinine concentration (mg/dL). 

Serum cystatin-C-based eGFRs were determined according to the following equations using serum cystatin C measured by four particle-enhanced immunoturbidimetric assays (PETIAs) [18–21] and one particle-enhanced immunonephelometric assay (PENIA) [22]:

1. eGFR = −6.87 + 87.1/Cys C (Tan et al. [18]),
2. eGFR = 99.19 × Cys C^−1.713^ × 0.823 (if female) (Grubb et al. [19]),
3. eGFR = −22.3 + 124/Cys C (Sjöström et al. [20]),
4. eGFR = 99.434 × Cys C^−1.5837^ (Larsson et al. [21]),
5. eGFR = −4.32 + 80.35/Cys C (Hoek et al. [22])

Cys C = serum cystatin C concentration (mg/L).

In this study, serum cystatin C was measured with a PETIA method. For a comparative reference, we also included eGFR calculated with the PENIA method by Hoek et al. because the Hoek formula showed the best overall performance for GFR estimation with respect to bias, precision, and accuracy, as compared with similar methods [22].

Data are expressed as mean ± SD. Changes in variables after the consumption of both beverages are expressed as means (95% CI). Statistical differences in variables between 5 habitual coffee consumers and 14 nonhabitual coffee consumers were examined using the Mann-Whitney test. Differences in most variables between coffee consumption and green tea consumption and those between before and at the end of the interventions were examined by paired *t*-test and repeated-measures ANOVA for normally distributed variables determined by the Kolmogorov-Smirnov test, respectively, or by the Wilcoxon rank test for nonnormally distributed variables. Correlations between changes in variables were examined by the Pearson correlation tests. Statistical analysis was performed using IBM-SPSS version 18.0 (SPSS Inc., Chicago, ILL, USA) and Statview version 5.0 (SAS Institute, Cary, NC, USA). Values of *P* < .05 were considered statistically significant.

## 3. Results

**Figure 1:**
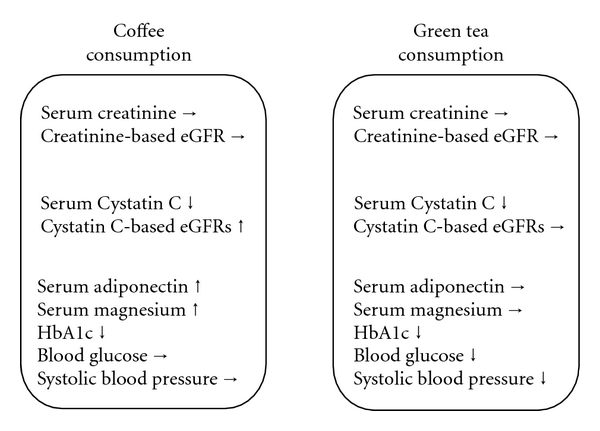
Summary of changes in variables according to coffee consumption and green tea consumption.

None of the subjects reported any adverse effects in response to consumption of coffee or green tea during the study, resulting in good adherence to the consumption of both beverages. The clinical characteristics of subjects and serum variables at baseline, after the 8-day washout period, are shown in Table 1. Overall, the subjects had favorable profiles for all characteristics that were within normal ranges. There were no significant differences in any of the variables between 5 habitual coffee consumers and 14 nonhabitual coffee consumers.  Table 2 shows the difference in variables between the start and the end of each intervention phase. Data before and after each treatment phase from two groups were pooled for each treatment. Serum creatinine levels were not altered after either intervention; also, creatinine-based eGFR was not affected by either beverage. In contrast, serum cystatin C was significantly decreased after coffee consumption (mean: −4.8%, *P* = .002, one-way repeated-measure ANOVA), but not after green tea consumption (mean: −1.3%). Serum adiponectin and magnesium levels increased after coffee consumption (means: 13.6% and 4.3%, resp.). By contrast, systolic blood pressure and blood glucose were significantly reduced by green tea consumption, whereas serum adiponectin and magnesium levels did not significantly increase after green tea consumption. Of note, a significant reduction in HbA1c was observed after both interventions.  Table 3 shows the difference in cystatin-C-based eGFRs calculated using five different equations between the start and the end of each intervention phase. Although cystatin-C-based eGFRs varied widely, all cystatin-C-based eGFRs increased significantly after coffee consumption (means: 5.0–7.7%), but not after green tea consumption (means: 0.1–1.6%). Nevertheless, the cystatin-C-based eGFRs at baseline before coffee consumption were significantly lower than those at baseline before green tea consumption (all *P* < .05). As shown in Table 4, the changes in cystatin-C-based eGFRs determined using five different equations were significantly correlated with changes in serum magnesium concentration after coffee consumption, but not after green tea consumption. There were no significant correlations between changes in serum cystatin-C and changes in other variables for both beverages (data not shown).  Figure 1 summarizes the difference in variables between the start and the end of each intervention phase, according to the beverage consumed.

## 4. Discussion

Coffee and green tea have been reported to have putatively protective effects against type 2 diabetes and cardiovascular diseases [2, 4, 5]. However, the underlying mechanisms remain unclear. To date, few studies have addressed the effects of these beverages or caffeine-containing beverages in general on kidney function [24], a key risk factor for cardiovascular diseases. In this study, we showed that coffee consumption for 2 weeks reduced cystatin-C and increased cystatin-C-based eGFR in apparently healthy young adults. Furthermore, the levels of serum adiponectin and magnesium were significantly increased after coffee consumption. Nevertheless, these effects of coffee consumption were not observed with green tea consumption.

Interestingly, these increases in eGFR after coffee consumption were not apparent after green tea consumption. This may be attributable to unintended bias because the eGFRs assessed using either creatinine and cystatin C were actually lower before coffee consumption than before green tea consumption. Nevertheless, we are unable to directly compare the effects of coffee consumption with those of green tea consumption because the green tea dose equivalent to coffee beans is not validated and needs to be determined in terms of kidney function. Furthermore, comparison of coffee with green tea per se was not our main purpose in this study.

Although the current findings suggest that coffee consumption seems to influence kidney function, the increased cystatin-C-based eGFR does not necessarily reflect improved kidney function. The subjects in this study had normal eGFR at baseline, and an increased eGFR might reflect glomerular hyperfiltration, which is often observed in obese people [25, 26] or at early stage of diabetic nephropathy [27]. To fully address whether the current findings are favorable and protective of kidney function, longitudinal prospective studies of coffee consumers are needed.

The increased adiponectin concentration after coffee consumption in the present study is consistent with previous reports [13, 14]. However, it is unlikely that the increased adiponectin levels directly contributed to the increase in eGFR because we found no significant correlation between changes in cystatin-C-based eGFR and changes in serum adiponectin levels. Furthermore, blood glucose and HbA1c were not associated with parameters related to kidney function, suggesting that changes in cystatin C-based eGFR may be independent of glucose metabolism. Nevertheless, the short duration of this study may conceal the intrinsic effects of coffee consumption on kidney function and clinical variables. 

The kidney is known to play a critical role in magnesium homeostasis and in maintaining plasma magnesium concentrations [28]. In this study, the increase in serum magnesium may reflect the absorption of nutrients present in coffee into the blood or increased magnesium reabsorption by the kidney. Several studies have reported inverse associations between serum magnesium concentrations and insulin resistance [29, 30]. Thus, although changes in cystatin C-based eGFRs were significantly correlated solely with those in serum magnesium in this study, it remains unclear whether the increased magnesium concentration directly modified the kidney function. It is possible that other bioactive constituents of coffee that were not examined in this study, such as major chlorogenic acid and phenolic acids, may contribute to this phenomenon. van Dijk et al. [31] provided evidence that chlorogenic acid and trigonelline reduced early glucose and insulin responses during a 2-hour oral glucose tolerance test in 15 overweight men. In addition, it is possible that chlorogenic acid improves postprandial glucose metabolism by reducing glucose uptake or increasing glucagon-like peptide-1 levels [32, 33]. This may explain why HbA1c, which reflects overall glucose homeostasis, but not blood glucose levels, was significantly improved following coffee consumption. However, in this study, HbA1c was also improved following green tea consumption, which is consistent with the findings of earlier studies [34, 35]. In this context, the mechanism by which coffee consumption improves HbA1c may differ from that involved in the effects of green tea.

Kempf et al. [14] reported that coffee consumption increased the concentrations of phenolic acids in the blood and reduced inflammatory markers, particularly in subjects with insulin resistance. Thus, these anti-inflammatory effects may also modify kidney function. Meanwhile, these possible mechanisms may be consistent with clinical findings that demonstrate the inverse relationship between coffee consumption and serum uric acid [36, 37], because serum uric acid is mostly excreted by the kidney.

Finally, caffeine, which is present in higher concentrations in a cup of coffee than in green tea [1], may also affect kidney function by antagonizing adenosine receptors and inhibiting phosphodiesterases [24, 38]. However, the acute effects of caffeine intake, particularly diuresis, are unlikely to be involved in the findings reported here because participants refrained from drinking coffee or green tea in the morning before laboratory test. Trials using decaffeinated coffee will reveal the effects of caffeine on eGFR.

Regarding the discrepancy between serum creatinine and serum cystatin C levels, the most plausible explanation is that serum cystatin C may be more sensitive to early changes in kidney function independently of muscle mass [10, 11]. However, in recent years, it has become acknowledged that the cystatin C level may be affected by factors other than kidney function, including sex, race, obesity, greater height, current cigarette smoking, thyroid function, inflammation, glucocorticoid function, and malignancy [9, 10, 39–41]. In this study, the subjects were young, nonobese, and nonsmokers without any critical diseases. Therefore, it is unlikely that these factors interfered with the outcomes of serum cystatin C, only after coffee consumption. However, the effects of coffee consumption on thyroid hormone and glucocorticoid hormone activities are still unclear. Furthermore, it is possible that kidney function as well as cystatin C metabolism (synthesis in cells and extrarenal elimination) might be changed by coffee consumption.

## 5. Limitations

First, we did not evaluate kidney function using standard GFR measurements, such as urinary clearance of insulin, the gold standard method, or two alternative protocols, namely urinary clearance of iothalamate and plasma clearance of iohexol [42]. Thus, precise evaluation of GFR is needed to determine the effects of coffee consumption on kidney function. Second, the change in cystatin C following coffee consumption was 0.04 (mg/L), which is small compared with the baseline cystatin C levels. However, the change in serum cystatin C was equivalent to changes in cystatin C-based eGFR of 4.7–10.3 mL/min/1.73 m^2^ (Table 3). Serum cystatin C appears to be a sensitive parameter that can detect mild GFR reductions of between 60 and 90 mL/min/1.73 m^2^ rather than moderate to severe reductions in GFR [9]. In addition, cystatin C has been evaluated for the early diagnosis of acute kidney injury [41]. Taken together, even small changes in serum cystatin C levels might reflect altered kidney function, although further studies are needed to elucidate the clinical relevance of such small changes in serum cystatin C. Third, the short duration of each intervention and the short washout period possibly interfered with the outcomes. Finally, because this trial was conducted in healthy young adults with normal eGFR, the findings may not be applicable to the general population or people with impaired kidney function or cardiovascular diseases.

## 6. Conclusion

We found that coffee consumption for 2 weeks decreased the serum cystatin C concentration and increased cystatin-C-based eGFR, along with favorable increases in serum adiponectin and magnesium concentrations in healthy young adults. Further studies with a larger study population, longer intervention time, longer washout periods, and measured GFR are needed to confirm the current findings and to address the underlying mechanisms.

## Figures and Tables

**Table 1:** Clinical characteristics of subjects.

	Mean ± SD
Age (years)	22.3 ± 1.7
Sex (male/female)	8/11
Height (cm)	163 ± 8.6
Body mass index (kg/m^2^)	21.3 ± 2.5
Systolic blood pressure (mm Hg)	120 ± 11.9
Diastolic blood pressure (mm Hg)	77 ± 8.8
Total cholesterol (mg/dL)	176 ± 21
High-density lipoprotein cholesterol (mg/dL)	68.7 ± 15.1
Triglycerides (mg/dL)	69.2 ± 37.5
Blood glucose (mg/dL)	85.6 ± 7.2
HbA1c (%, NGSP)	5.1 ± 0.2
HbA1c (mmol/mol, IFCC)	37 ± 2
Uric acid (mg/dL)	5.2 ± 1.3
Blood urea nitrogen (mg/dL)	14.5 ± 3.2
Creatinine (mg/dL)	0.71 ± 0.17
Creatinine-based eGFR (mL/min per 1.73 m^2^)	101 ± 16.5
Cystatin C (mg/L)	0.81 ± 0.13
Magnesium (mg/dL)	2.3 ± 0.2
Adiponectin (*μ*U/mL)	10.6 ± 4.7
Habitual coffee consumption, *n* (%)	5 (26.3)
Habitual tea consumption, *n* (%)	7 (36.8)
Habitual alcohol consumption, *n* (%)	3 (15.8)

NGSP; National Glycohemoglobin Standardization Program, IFCC; International Federation of Clinical Chemistry and Laboratory Medicine.

Habitual coffee, green tea, and alcohol consumers were defined as subjects who drink one or more cups of coffee or green tea and those who drink alcohol everyday, respectively.

**Table 2:** Changes in variables according to coffee and green tea consumption.

	Coffee			Green tea		
	Baseline	End of consumption	Change (95% CI)	Baseline	End of consumption	Change (95% CI)
BMI (kg/m^2^)	21.2 ± 2.5	21.3 ± 2.4	0.1 (0.0–0.2)	21.4 ± 2.5	21.2 ± 2.5	−0.2 (−0.3–0.0)
SBP (mm Hg)	118 ± 16	119 ± 11	0.7 (−5.9–7.4)	121 ± 11	116 ± 9.3*	−0.5 (−9.6–−0.3)
DBP (mm Hg)	75 ± 8.8	77 ± 9.1	2.5 (−1.2–6.2)	78 ± 8.9	76 ± 7.4	−1.7 (−6.9–3.4)
Total cholesterol (mg/dL)	174 ± 22	175 ± 26	1.7 (−5.1–8.6)	176 ± 19	170 ± 22	−5.4 (−12.5–1.7)
HDL-cholesterol (mg/dL)	66 ± 15	67 ± 14	0.6 (−2.6–3.7)	65 ± 13	66 ± 13	0.4 (−2.2–3.1)
Triglycerides (mg/dL)	70 ± 33	86 ± 52	16.4 (−2.8–35.7)	79 ± 38	72 ± 46	−6.3 (−25.5–12.9)
Blood glucose (mg/dL)	84 ± 10	84 ± 9.4	0.1 (−5.1–5.3)	87 ± 5.6	84 ± 7.9*	−3.4 (−6.8–−0.1)
HbA1c (%, NGSP)	5.1 ± 0.2	5.0 ± 0.2^∗,a^	−0.1 (−0.1–0.0)	5.1 ± 0.2	5.0 ± 0.2*	−0.1 (−0.1–0.0)
Uric acid (mg/dL)	5.2 ± 1.2	5.2 ± 1.1	0.1 (−0.2–0.3)	5.1 ± 1.1	5.1 ± 1.3	0.0 (−0.3–0.2)
Blood urea nitrogen (mg/dL)	14.3 ± 3.3	13.6 ± 2.7	−0.7 (−2.1–0.7)	13.6 ± 3.4	13.1 ± 3.2	−0.5 (−2.1–1.2)
Serum creatinine (mg/dL)	0.72 ± 0.16^†^	0.72 ± 0.17	0.00 (−0.02–0.02)	0.70 ± 0.15	0.71 ± 0.17	0.01 (−0.01–0.02)
Creatinine based-eGFR (mL/min/1.73 m^2^)	99.6 ± 16.4	99.6 ± 16.6	0.0 (−3.4–3.4)	102.0 ± 15.9	102.0 ± 19.0	0.1 (−2.9–3.0)
Serum cystatin C (mg/L)	0.83 ± 0.12^†^	0.79 ± 0.10**	−0.04 (−0.06–−0.02)^ †^	0.79 ± 0.12	0.79 ± 0.12	−0.01 (−0.03–0.02)
Magnesium (mg/dL)	2.3 ± 0.1	2.4 ± 0.2^∗,†^	0.1 (0.0–0.1)	2.3 ± 0.1	2.3 ± 0.2	0.0 (0.0–0.1)
Adiponectin (*μ*g/mL)	11.0 ± 4.7	12.5 ± 5.3**	1.5 (0.6–2.4)	10.5 ± 4.6	11.7 ± 5.0	1.1 (−0.6–2.9)

Data are expressed as means ± SD. Changes in variables are expressed as means (95% CI).

*P < .05, **P < .01, End of consumption versus baseline (repeated ANOVA), ^†^ *P* < .05, versus green tea (Paired *t*-test).

a difference was examined by Wilcoxon rank test because normal distribution was not confirmed.

BMI: body mass index, SBP: systolic blood pressure, DBP: diastolic blood pressure, HDL: high-density lipoprotein, eGFR: estimated glomerular filtration rate.

**Table 3:** Difference in cystatin-C-based eGFR (determined using five different equations) between the start and end of each consumption period.

Cystatin-C-based eGFR		Coffee				Green tea			
(mL/min/1.73 m^2^)		Baseline	End of consumption	*P* value	Change (95% CI)	Baseline	End of consumption	*P* value	Change (95% CI)
Tan et al. [18]	PETIA	101 ± 15.8^†^	106 ± 14.4	.01	5.1 (1.3–8.9)^†^	105 ± 17.1	106 ± 17.9	.51	1.1 (−2.4–4.6)
Grubb et al. [19]	PETIA	128 ± 27.0^†^	137 ± 21.6	.02	9.9 (1.8–18.0)	137 ± 28.6	139 ± 31.3	.50	2.4 (−5.0–9.9)
Sjöström et al. [20]	PETIA	131 ±22.5^†^	138 ± 20.4	.01	7.2 (1.8–12.7)^†^	137 ± 24.4	139 ± 25.5	.51	1.6 (−3.4–6.5)
Larsson et al. [21]	PETIA	140 ± 33.1^†^	150 ± 30.2	.02	10.3 (1.8–18.8)^†^	150 ± 36.0	152 ± 38.3	.50	2.4 (−5.1–10.0)
Hoek et al. [22]	PENIA	94.9 ± 14.6^†^	99.5 ± 13.2	.01	4.7 (1.1–8.2)^†^	99.2 ± 15.8	100 ± 16.5	.51	1.0 (−2.1–4.2)

*P* values were determined by repeated-measure ANOVA. ^†^ *P* < .05 versus green tea (paired *t* test).

**Table 4:** Correlation coefficients between changes in cystatin-C-based eGFR (determined using five different equations) and changes in serum magnesium between the start and end of each consumption period.

Cystatin C-based eGFR	Coffee		Green tea	
	*r*	*P* value	*r*	*P* value
Tan et al.	0.47	.04	0.08	.75
Grubb et al.	0.48	.04	0.10	.68
Sjöström et al.	0.47	.04	0.08	.75
Larsson et al.	0.49	.03	0.09	.75
Hoek et al.	0.47	.04	0.08	.75

Correlation coefficients were examined by the Pearson correlation tests.
